# Valproic Acid Protects Chondrocytes from LPS-Stimulated Damage via Regulating miR-302d-3p/ITGB4 Axis and Mediating the PI3K-AKT Signaling Pathway

**DOI:** 10.3389/fmolb.2021.633315

**Published:** 2021-04-22

**Authors:** Long Sun, Wei Zheng, Qian-Dong Liu, Lei Ge

**Affiliations:** ^1^ Department of Orthopedics, Weihai Municipal Hospital, Cheeloo College of Medicine, Shandong University, Weihai, China; ^2^ Department of Joint Surgery, Rizhao Central Hospital, Rizhao, China; ^3^ Department of Emergency, People’s Hospital of Rizhao, Rizhao, China

**Keywords:** osteoarthritis, valproic acid, autophagy, miR-302d-3p/ITGB4, PI3K-Akt pathway

## Abstract

**Background:** Osteoarthritis (OA) is one of the most common degenerative joint diseases characterized by increased apoptosis and autophagy deficiency. The investigation was performed to examine the effect of valproic acid (VPA) and molecular mechanism related to miR-302d-3p/ITGB4 axis in OA.

**Methods:** The OA clinical samples were obtained from the GEO database to analyze differentially expressed genes. An *in vitro* OA model was mimicked by LPS in CHON-001 cells. Autophagy-related genes were downloaded from the HADb website, and potential drugs were mined using the CTD website. The upstream factors of ITGB4 were predicted with bioinformatics analysis, which was validated by luciferase activity assay and RIP assay. Cell viability and apoptosis were evaluated using CCK-8 and flow cytometry. The expression levels, including ITGB4, miR-302d-3p, and autophagy-/PI3K-AKT pathway-related markers, were measured by qRT-PCR or/and western blot.

**Results:** Our results showed that miR-302d-3p inhibited cell viability and promoted apoptosis of LPS-treated CHON-001 cells by targeting ITGB4. VPA treatment remarkably alleviated LPS-stimulated injury in CHON-001 cells. The inhibitory effect of VPA on LPS-stimulated damage in CHON-001 cells was weakened by miR-302d-3p overexpression, while it was intensified because of ITGB4 upregulation. Mechanistically, VPA treatment induced a significant decrease in the levels of p-PI3K and p-AKT in LPS-stimulated CHON-001 cells through regulating miR-302d-3p/ITGB4 axis.

**Conclusion:** Overall, VPA treatment may ameliorate LPS-induced injury on chondrocytes via the regulation of miR-302d-3p/ITGB4 pair and the inactivation of the PI3K-AKT pathway.

## Background

Osteoarthritis (OA) is one of the prevalent chronic degenerative joint diseases, whose symptoms mainly include joint swelling and long-term chronic pain (Hawker, 2019). The causative factors are aging, obesity, previous joint injury, sex, genetics, and some anatomical factors related to joint shape or alignment (Shen et al., 2019). The pathogenesis of OA is unclear, and effective strategies are not available to retard its development (Si et al., 2020). Hence, considerable attention has been focused on the identification of novel biomarkers and efficacious interventions for OA.

Autophagy is a lysosomal-dependent degradation reaction. It maintains organelle and protein functions and attenuates the detrimental stress during cell apoptosis (Choi et al., 2013). Existing evidence has suggested that autophagy is suppressed in OA (Shapiro et al., 2014; Zhang et al., 2015) and recovers the function of injured chondrocytes (Duan et al., 2020). According to our screening work, integrin β4 (ITGB4) was identified as a differentially expressed gene (DEG) in OA and is related to autophagy. Integrins are composed of noncovalent associations of *α* and *ß* dimmers, playing important roles in the extracellular matrix (ECM) (Hynes, 2002). ITGB4, as a member of integrins, heterodimerizes with integrin subunit α6 (ITGA6) (Stewart and O’Connor, 2015). Several studies have demonstrated that ITGB4 is involved in metastasis and tumorigenesis of breast cancer, gastric cancer, and lung cancer (Lipscomb et al., 2005; Nagata et al., 2013; Yang et al., 2013). The research studies on the association of ITGB4 in OA, however, still remain rare.

miRNAs are a class of multifunctional short noncoding RNA molecules implicated in various biological and pathological processes by directly binding to the 3′-UTR of the target genes (Lu et al., 2016). Increasing evidence suggests that several miRNAs are related to the progression of OA via modulating autophagy in chondrocytes (Xiao et al., 2020; Zhang et al., 2020). Bioinformatics analysis of our study manifested that miR-302d-3p may directly target ITGB4. miR-302d-3p is a member of the miR-302 cluster that was initially found in human embryonic stem cells, which plays a crucial role in endometrial carcinoma (Li et al., 2018), cervical squamous cell cancer (Wang and Chen, 2020), and breast cancer (Sun et al., 2020a). More importantly, Wang et al. (2019) have indicated that miR-302d-3p is expressed at higher levels in OA and may hinder the progression of OA via mediating cell viability in chondrocytes. Nevertheless, the underlying molecular mechanism of miR-302d-3p and ITGB4 in OA is unknown.

PI3K-AKT pathway is an intracellular pathway and correlated with cell proliferation, motility, tumor, and longevity (Carnero et al., 2008). It has been indicated as a fated event for maintaining joint health and mediating the progression of OA (Sun et al., 2020b). The activation of the PI3K-AKT signaling pathway could accelerate the development of OA (Xue et al., 2017). Additionally, ITGB4 was enriched in the PI3K-AKT pathway based on bioinformatics analysis. Thus, we speculated that the PI3K-AKT pathway might be involved in the regulatory action of the ITGB4/miR-302d-3p axis in OA.

With the help of the CTD website, valproic acid (VPA) was identified as a putative agent associated with OA progression, autophagy, and ITGB4 expression. VPA is one such flavonoid extracted from the herbaceous plant and a first-line agent that is used to treat epilepsy and manic disease (Henry, 2003). VPA has been shown to exert multiple therapeutic actions, including anticancer, neuroprotection, differentiation, and neuroregeneration activities (Abematsu et al., 2010; Morris and Monteggia, 2013). VPA functions as an inducer for epigenetic changes and inhibits miRNAs expression (Jessberger et al., 2007; Hunsberger et al., 2012). Of note, VPA could induce the osteogenesis of mouse mesenchymal stem cells (Akshaya et al., 2021). But, the potential effect of VPA on OA has yet to be investigated.

In the present study, therefore, we examined the effects of the VPA and miR-302d-3p/ITGB4 axis in LPS-treated chondrocytes. The regulatory action of VPA on ITGB4 and miR-302d-3p expression was also explored. The regulatory mechanism related to VPA/miR-302d-3p/ITGB4 may support the experimental basis for VPA’s clinical application and provide the new biomarkers for the treatment of OA.

## Methods

### Cell and Osteoarthritis *In Vitro* Model

Human chondrocyte CHON-001 cells were purchased from Procell Life Science & Technology Co., Ltd. (Wuhan, China) and incubated in the Dulbecco’s modified Eagle’s medium (DMEM; Invitrogen, Carlsbad, CA, United States), containing 10% FBS, 100 units/ml penicillin, and 100 mg/ml streptomycin under 5% CO_2_ at 37°C. CHON-001 cells were stimulated with LPS (0, 1, 2.5, 5, and 10 µg/ml) for 12 h to construct an OA *in vitro* model (Hu and Li, 2019; Sui et al., 2019).

### Chemical Reagent Treatment

Valproic acid (VPA; Sigma-Aldrich) stock solution was dissolved with DMSO and diluted in the culture medium to the final test concentrations (0, 0.5, 1, 2, and 5 mM) for CHON-001 cell incubation.

### miRNA, Plasmid Construction, and Transfection

Cells with over 80% confluence were seeded into a six-well plate and transfected with negative control (NC, 5′-CTC​CCT​TCT​CTT​CTC​CCG​TCT​T-3′), miR-302d-3p mimic (5′-TAAGTGCTTCCATGTTTGAGTGT-3′)/inhibitor (5′-ACA​CTC​AAA​CAT​GGA​AGC​ACT​TA-3′), scramble siRNA (si-con, 5′-GAT​CGT​TCC​AGT​ACG​AAG​TCA​TGG-3′), si-ITGB4 (5′-GAG​GGT​GTC​ATC​ACC​ATT​GAA​CTC-3′), and pcDNA3.1-ITGB4 vector using Lipofectamine 2000. All the abovementioned bioagents were provided by GenePharma Co. (Shanghai, China). Chondrocytes were then cultured 48 h for future investigation in the presence or absence of VPA.

### Real-Time Quantitative Polymerase Chain Reaction

Total RNA of transfected chondrocytes was extracted with the TRIZOL reagent (Invitrogen, Carlsbad, CA, United States). Oligo (dT) primers were utilized to conduct the reverse transcription. The real-time quantitative PCR assay was performed with TaqMan one-step PCR Master Mix (Applied Biosystems, Foster City, CA, United States) and SYBR Premix Ex Taq (Invitrogen) on ABI7900HT real-time PCR system. For miRNA, the cDNA was synthesized by the TaqMan MicroRNA reverse transcription kit (Applied Biosystems). All reaction conditions were set up as follows: 95°C for 10 min, then 39 cycles consisting of predenaturation at 95°C for 10 s, denaturation at 60°C for 45 s, and extension at 72°C for 30 s, followed by final extension at 72°C for 5 min. Relative expression levels were calculated using the 2^−ΔΔCt^ method, with specific controls, GAPDH for ITGB4 and U6 for miR-302d-3p.

Primers were listed as follows:

  miR-302d-3p
  F: 5′-TGC​TTC​CAT​GTT​TGA​GTG​TG -3′,
  R: 5′-GAA​CAT​GTC​TGC​GTA​TCT​C -3’;
  U6
  F: 5′-CTC​GCT​TCG​GCA​GCA​CA -3′,
  R: 5′-AAC​GCT​TCA​CGA​ATT​TGC​GT -3’;
  ITGB4
  F: 5′-AGG​ATG​ACG​ACG​AGA​AGC​AGC​T-3′,
  R: 5′-ACC​GAG​AAC​TCA​GGC​TGC​TCA​A-3’;
  GAPDH
  F: 5′-CCA​CAG​TCC​ATG​CCA​TCA​CT-3′,
  R: 5′-AGT​GAT​GGC​ATG​GAC​TGT​GG-3’.

### Western Blotting

5 µg/ml LPS was used to treat CHON-001 cells for 5 h after 48 h transfection. Protein extraction was then implemented in RIPA buffer (Beyotime, Beijing, China) supplemented with 1% protease inhibitor cocktail. The quantification of proteins was detected with the BCA method, and separated proteins were denatured at 95°C for 5 min. Electrophoresis was conducted in 12% SDS-PAGE with an equal amount of protein (20 μg) per tank, and the protein bands were transferred onto PVDF membranes. Afterward, these membranes were sealed in 5% skimmed milk powder for 60 min and incubated with primary antibodies at 4°C overnight. After rising in TBST, PVDF membranes were incubated with secondary antibody at ambient temperature for 60 min. All antibodies were as follows: Beclin 1 (cat. no. 3738; 1:1,000, CST), LC3-I/II (cat. no. 23214; 1:1,000, CST), p62 (cat. no. 5114; 1:1,000, CST), p-PI3K (cat. no. 4228; 1:1,000, CST), PI3K (cat. no. 4292; 1:1,000, CST), p-AKT (cat. no. 9271; 1:1,000, CST), AKT (cat. no. 9272; 1:1,000, CST), ITGB4 (cat. no. 4707; 1:1,000, CST), GAPDH (cat. no. 8884; 1:1,000, CST), and the HRP-conjugated anti-rabbit secondary antibody (cat. no. 7074; 1:2,000, CST). Protein signals were developed with ECL (EMD Millipore), and all immunoblots were scanned by Quantity One software (Bio-Rad, Hercules, CA, United States). The results of two repeat western blot assays were showed in Supplementary Figure 1.

### CCK-8 Assay

Cell viability was assessed by using a CCK-8 kit (Takara, Japan) according to the manufacturer’s protocols. Transfected chondrocytes exposed to VPA or not were firstly seeded into a 96-well plate. After 0, 24, 48, and 72 h incubation, 10 µL of CCK-8 cocktail was added to each well to continuously maintain cells for other 1.5 h at 37°C with 5% CO_2_. The absorbance values were measured at 450 nm under a microplate reader.

### Apoptosis Analysis

The proportion of apoptosis was examined with the Annexin V-FITC/PI double staining kit. Chondrocytes (2 × 10^5^) were collected in a 5 ml tube and centrifuged at 2,000 xg at 4°C. The supernatant was discarded, and cells were resuspended using 1× binding buffer. Next, 100 µL of supernatant, 5 µL of Annexin V-FITC, and 5 µL of PI/RNase were mixed in tubes and incubated in the dark at room temperature for 5 min. Finally, 100 µL of PBS was added and flow cytometry machine detection was performed within 1 h. The FITC channel screens GFP-positive cells for Annexin V-APC/7-AAD fluorescence detection by GFP green fluorescence. The red fluorescence of Annexin V-APC was viewed at an excitation wavelength of 633 nm and a maximum emission wavelength of 660 nm. The excitation wavelength was 546 nm, and the emission wavelength was 647 nm for the 7-AAD red fluorescence. Single-color stains for FITC Annexin and PI and unstained cells were included in all experiments as positive and negative controls. The quadrants were as follows: Q1, necrotic cells; Q2, late apoptotic cells; Q3, viable cells; Q4, apoptotic cells.

### Luciferase Activity Assay

Based on the bioinformatics analyses on the TargetScan, StarBase, and miRDB, the binding sites were predicted between miR-302d-3p and ITGB4. To validate the correlation, the 3′-UTR of ITGB4 sequences was amplified and inserted into the pLUC vector to construct the ITGB4 wild type (WT). Similarly, ITGB4 mutant (MUT) was generated by the same steps with mutant sequences of binding sites. In order to examine the luciferase activity, CHON-001 cells were transfected with ITGB4 WT or ITGB4 MUT and miR-NC, miR-302d-3p mimic, or inhibitor using Lipofectamine 2000. After 48 h transfection, lysis buffer was used to lyse cells, and the Renilla and Firefly luciferase activities were measured using a Dual-Luciferase Reporter Assay kit (Promega Corporation, Madison, WI, United States). The ratio of Firefly luciferase activity to Renilla luciferase activity was utilized to reflect the association between miR-302d-3p and ITGB4.

### RNA Immunoprecipitation Assay

The interaction between miR-302d-3p and ITGB4 was further ascertained using Magna RIPTM RNA Binding Protein Immunoprecipitation Kit (Millipore, Bedford, MA, United States). CHON-001 cells transfected with miR-NC or miR-302d-3p mimic were lysed in precooled RIP buffer. The cell lysate was then hatched with Protein A/G magnetic beads and continued to be incubated with anti-Ago2 (Abcam) or anti-IgG (Abcam). After washing, the protein and DNA in the immunoprecipitated complex were removed and qRT-PCR was conducted to evaluate the enrichment level of ITGB4.

### Data Processing and Statistics

OA clinical specimens, accession numbers GSE55457 and GSE12021, were collected from the GEO database and used for the identification of DEGs. Autophagy-related genes were downloaded from the HADb repository (http://www.autophagy.lu/). KEGG analysis was performed on the DAVID website. Drugs related to OA were determined with the CTD website. Statistical analysis was applied with SPSS19.0 and GraphPad Prism 8.0, and comparisons were determined using Student’s *t*-test or ANOVA followed by Tukey’s post hoc test. All variables were presented as mean ± standard deviation (SD), which were obtained from three independent experiments. *p* < 0.05 indicates significant difference.

## Results

### Identification of Potential Biomarkers and Agents in Osteoarthritis

**FIGURE 1:**
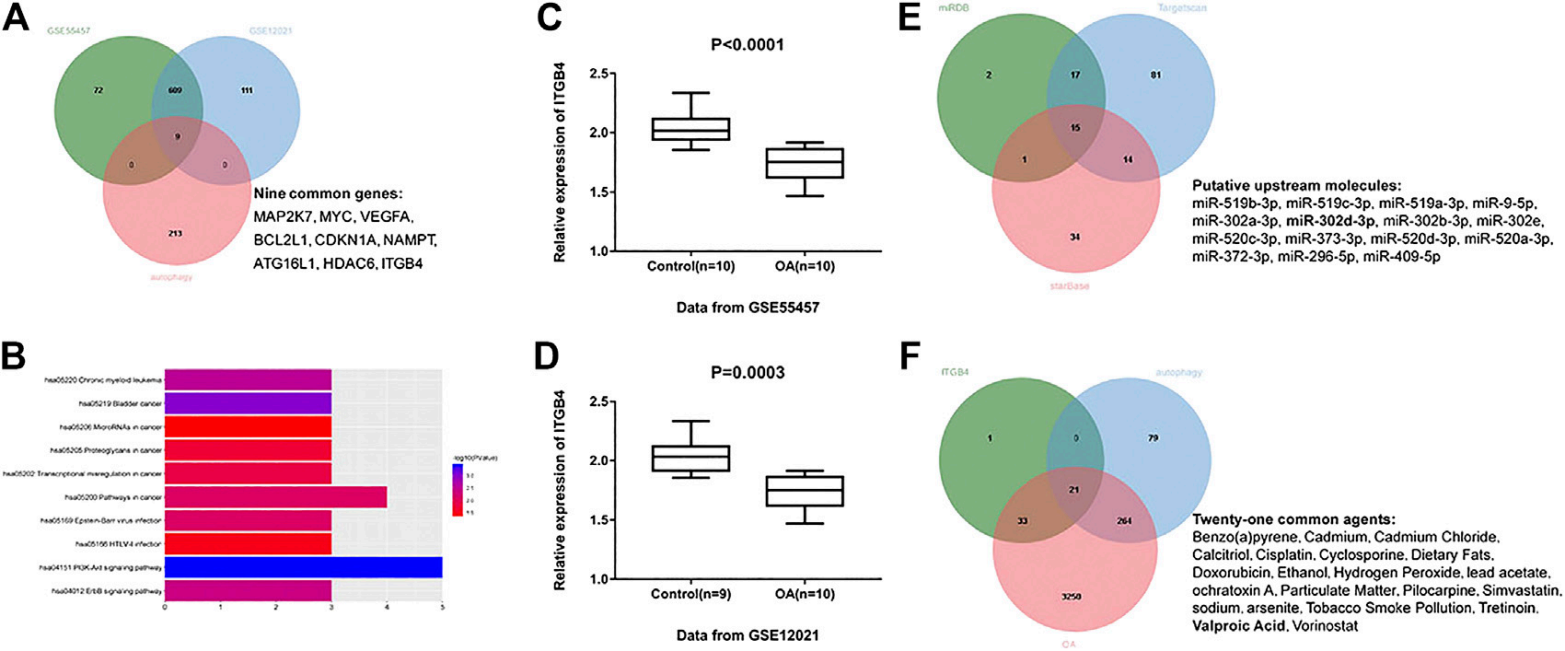
Identification of potential biomarkers and agents in OA. **(A)** Intersection of differentially expressed genes identified based on GSE55457, GSE12021, and autophagy-related genes. Green indicates differentially expressed genes based on GSE55457; blue indicates differentially expressed genes based on GSE12021; red indicates autophagy-related genes downloaded from HADb websites. **(B)** The top ten meaningful signaling pathways were identified by analyzing the above nine common genes in the DAVID website for KEGG pathway enrichment analysis. **(C)** Relative expression of ITGB4 was decreased in OA tissues (*n* = 10) compared with normal controls (*n* = 10) on the basis of GSE55457. *p* < 0.0001. **(D)** GSE12021 dataset showed a decreased ITGB4 level in OA tissue samples (*n* = 10) compared with controls (*n* = 9). *p* = 0.0003. **(E)** miR-302d-3p was identified as a putative upstream factor of ITGB4, according to the bioinformatics prediction. Green represents potential miRNAs using miRDB; blue represents potential miRNAs using TargetScan; red represents potential miRNAs using starBase. **(F)** VPA was screened out as a potential drug related to OA progression, autophagy, and ITGB4 expression. Green indicates potential drugs related to ITGB4 expression; blue indicates autophagy-related drugs; red indicates OA-related drugs. Data were expressed as the mean ± SD (Student’s *t*-test).

To clearly explore the pathogenesis of OA, OA clinical samples retrieved from the GEO database (accession numbers: GSE55457 and GSE12021) were utilized to identify the DEGs in OA according to the following criteria: log (fold change) ≥1 and *p* < 0.05. A total of 690 DEGs were identified based on GSE55457, including 211 upregulated mRNAs and 479 downregulated mRNAs. Meanwhile, a total of 729 DEGs (212 upregulated genes and 517 downregulated genes) were achieved on the basis of GSE12021. Afterward, we accessed the HADb repository and downloaded 222 autophagy-related genes. In the overlapping part of 222 autophagy-related genes and DEGs achieved from GSE55457 and GSE12021, nine common genes were obtained (Figure 1A), including MAP2K7, MYC, VEGFA, BCL2L1, CDKN1A, NAMPT, ATG16L1, HDAC6, and ITGB4. These nine common genes were analyzed in the DAVID database for KEGG enrichment analysis and ten meaningful pathways were obtained under the condition of *p* < 0.05 (Figure 1B). The pathway with the most enriched genes was the PI3K-AKT signaling pathway. For genes enriched in the PI3K-AKT signaling pathway, a review of related articles demonstrated that only ITGB4 has yet to be studied in OA. Thus, we finally selected ITGB4 as a research objective. As shown in Figures 1C,D, the expression of ITGB4 in OA tissues was remarkably lower than normal controls.

Next, we employed TargetScan, StarBase, and miRDB prediction tools to predict the upstream miRNAs of ITGB4. We took the intersection of potential miRNAs obtained from these three prediction sites and ultimately obtained 15 candidate miRNAs (Figure 1E). Given the previous literature, except for miR-302d-3p and miR-373-3p, other miRNAs of ITGB4 were not studied in OA (Wang et al., 2019; Zhu and Jiang, 2019). Considering that ITGB4 was decreased in OA, we finally selected miR-302d-3p with high expression in OA (Li et al., 2018).

The CTD website was subsequently used to query the drugs related to OA, autophagy, and ITGB4 expression, owing to the lack of effective agents in OA. A total of 21 common drugs were identified (Figure 1F). Through comprehensive literature analysis, VPA was selected for further research. Accordingly, we realized that miR-302d-3p and ITGB4 may be crucial therapeutic targets for OA treatment and hypothesized that VPA might regulate the progression of OA through mediating miR-302d-3p/ITGB4 signal axis.

### Valproic Acid Ameliorates LPS-Induced Injury in CHON-001 Cells

**FIGURE 2:**
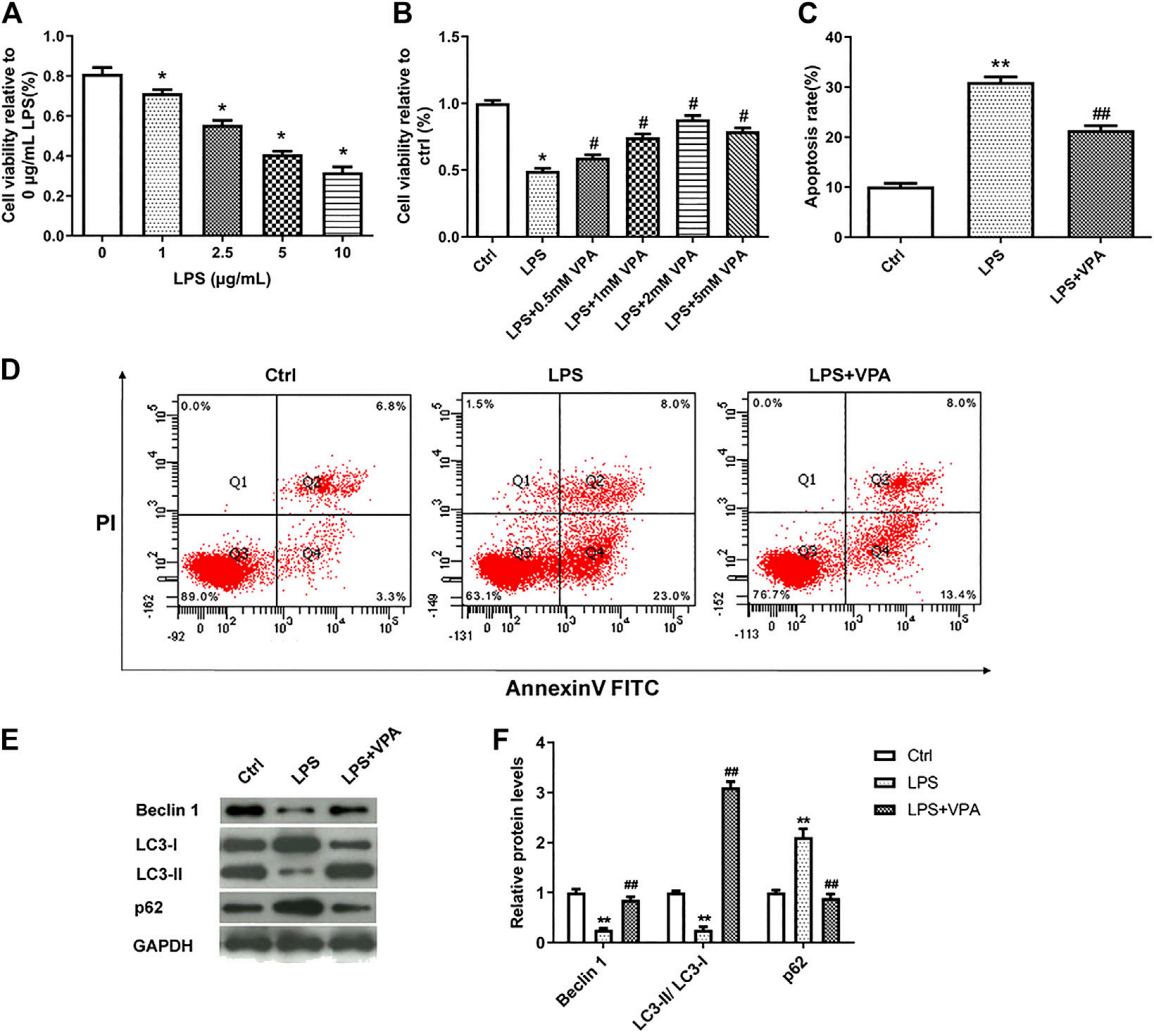
VPA ameliorates LPS-induced injury in CHON-001 cells. **(A)** The effect of LPS on the cell viability in chondrocytes using CCK-8 kit. *p < 0.05. **(B)** VPA treatment reversed the cell viability of LPS-induced CHON-001 cells, based on the CCK-8 assay results. *p < 0.05, ^#^ *p* < 0.05. **(C,D)** The apoptosis rate of chondrocytes was detected with Annexin V-FITC/PI double staining assay. **p < 0.01, ^##^ *p* < 0.01. **(E,F)** Protein levels of Beclin 1, LC3-I, LC3-II, and p62 were assessed using western blotting and quantified. **p < 0.01, ^##^ *p* < 0.01. The average of three independent experiments in triplicate is shown with error bars indicating mean ± SD (ANOVA followed by Tukey’s post hoc test).

To validate the hypothesis, we firstly mimic human OA using LPS-treated CHON-001 cells. CCK-8 assay revealed that LPS inhibited cell viability in a dose-dependent manner (Figure 2A). 5 µg/ml LPS was selected for further experiments. Next, the effect of VPA on cell viability was examined by CCK-8 assay. Results in Figure 2B manifested that the treatment of VPA (0, 0.5, 1, 2, and 5 mM) significantly relieved the LPS-stimulated injury in CHON-001 cells, and the protective effect reached its peak at 2 mM; thus, we selected 2 mM for further experiments. LPS treatment induced an increased apoptosis rate in CHON-001 cells, which was attenuated after VPA treatment (Figures 2C,D). The levels of autophagy-related markers (Beclin 1, LC3-I, LC3-II, and p62) were measured using western blot. Results revealed that the level of Beclin 1 and the conversion of LC3-I to LC3-II were inhibited in LPS-treated CHON-001 cells, and p62 was significantly increased (Figures 2E,F). Therefore, we speculated that autophagy was suppressed in LPS-treated chondrocytes. The VPA stimulation overturned the suppressive effect of LPS on the autophagy of chondrocytes (Figures 2E,F). Overall, these findings suggested that VPA might protect chondrocytes against LPS-induced injury.

### miR-302d-3p/ITGB4 Axis Modulates LPS-Treated CHON-001 Cell Viability and Apoptosis

**FIGURE 3:**
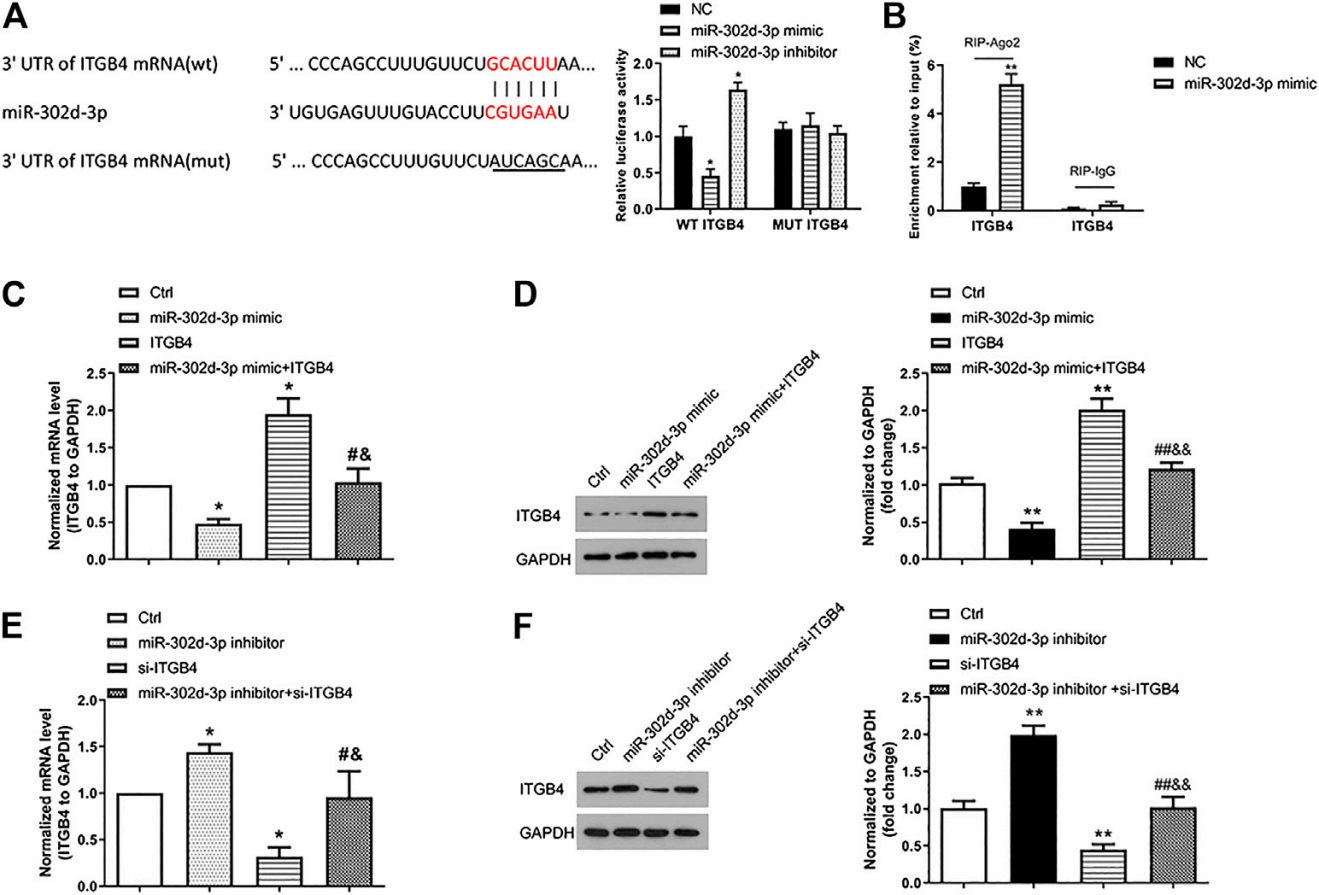
ITGB4 is a direct target gene of miR-302d-3p. **(A)** Sequence alignment between miR-302d-3p and 3′-UTR ITGB4. A dual-luciferase reporter gene assay was performed to verify the relationship between them. *p < 0.05. **(B)** RIP assay was utilized to examine the enrichment degree of ITGB4 in IgG or Ago2 immunoprecipitation complex. **p < 0.01. **(C,D)** Effect of miR-302d-3p mimic, pcDNA3.1-ITGB4, and miR-302d-3p mimic + pcDNA3.1-ITGB4 on the expression of ITGB4 in CHON-001 cells using qRT-PCR and western blotting experiments. *p < 0.05, ^#^ *p* < 0.05, ^&^ *p* < 0.05. **(E,F)** Impact of miR-302d-3p inhibitor, si-ITGB4, and miR-302d-3p inhibitor + si-ITGB4 on the expression of ITGB4 in CHON-001 cells was examined with qRT-PCR and western blot assays. *p < 0.05, ^#^ *p* < 0.05, ^&^ *p* < 0.05. Results represent the mean of three independent experiments. Errors bars indicated mean ± SD (ANOVA followed by Tukey’s post hoc test).

The biological function of miR-302d-3p/ITGB4 axis on LPS-treated chondrocytes was conducted in the following experiments. Before that, we first verified the relationship of ITGB4 and miR-302d-3p. The complementary region sequences between 3′-UTR ITGB4 and miR-302d-3p are presented in Figure 3A. Accordingly, the luciferase reporter vectors ITGB4 WT and ITGB4 MUT were constructed and utilized to evaluate the luciferase activity using a dual-luciferase report assay. Relative luciferase activity of the ITGB4 WT group was significantly decreased after the transfection of miR-302d-3p mimic, whereas miR-302d-3p inhibitor resulted in an increased level of luciferase activity. However, there were no changes in the ITGB4 MUT group (Figure 3A). To further ascertain the interaction between miR-302d-3p and ITGB4, RIP assay was also implemented. Results showed that overexpression of miR-302d-3p induced the copious enrichment of ITGB4 in the Ago2 immunoprecipitation complex, indicating the target interaction between miR-302d-3p and ITGB4 (Figure 3B). The interplay of miR-302d-3p and ITGB4 was analyzed through qRT-PCR and western blot. The expression of ITGB4 was inhibited by miR-302d-3p overexpression but was promoted after miR-302d-3p knockdown (Figures 3C–F). CHON-001 cells transfected with pcDNA3.1-ITGB4 showed an increased ITGB4 expression but a decreased level of ITGB4 after si-ITGB4 transfection (Figures 3C–F). Besides, as expected, the promoting effect of pcDNA3.1-ITGB4 or inhibitory impact induced by si-ITGB4 on ITGB4 expression was attenuated due to the transfection of miR-302d-3p mimic or inhibitor, respectively (Figures 3C–F). Collectively, all results demonstrated that ITGB4 may be directly targeted by miR-302d-3p.

**FIGURE 4:**
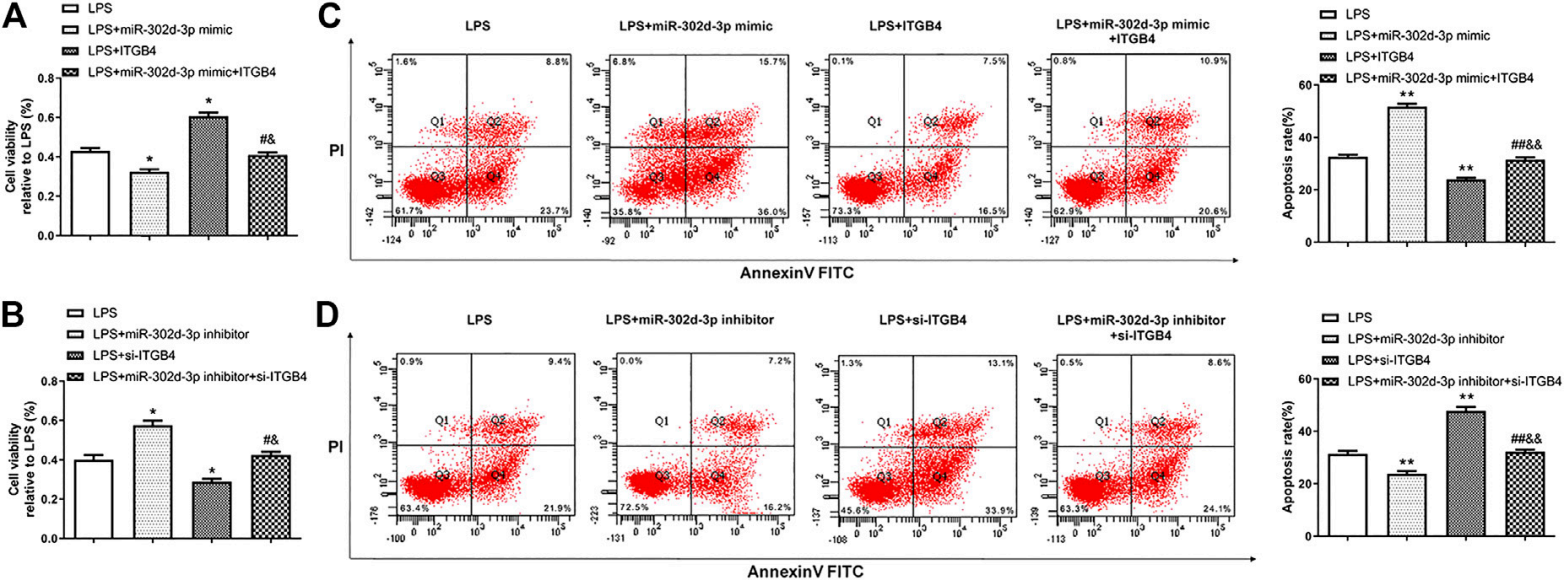
miR-302d-3p/ITGB4 axis modulates LPS-treated CHON-001 cells viability and apoptosis. **(A,B)** CCK-8 assay was utilized to evaluate cell viability of LPS-treated CHON-001 cells. *p < 0.05, ^#^ *p* < 0.05, ^&^ *p* < 0.05. **(C,D)** Flow cytometry assay was used to measure the apoptosis rate of each group. **p < 0.01, ^##^ *p* < 0.01, ^&&^ *p* < 0.01. Results are the average of three individual experiments in triplicate with error bars indicating mean ± SD (ANOVA followed by Tukey’s post hoc test).

Next, CCK-8 assay revealed that miR-302d-3p mimic or si-ITGB4 dramatically inhibited the cell viability of LPS-treated CHON-001 cells (Figures 4A,B). Conversely, cell viability of CHON-001 cells stimulated by LPS was elevated after the transfection of miR-302d-3p inhibitor or pcDNA3.1-ITGB4 (Figures 4A,B). Moreover, the cotransfection of miR-302d-3p mimic and pcDNA3.1-ITGB4 or miR-302d-3p inhibitor and si-ITGB4 reversed the corresponding impacts of individual transfection of miR-302d-3p mimic, pcDNA3.1-ITGB4, miR-302d-3p inhibitor, and si-ITGB4 (Figures 4A,B). The opposite trends were exhibited in flow cytometry assays. In LPS-stimulated chondrocytes, miR-302d-3p overexpression significantly promoted apoptosis rate, while upregulation of ITGB4 reduced the apoptosis rate. Cotransfection of miR-302d-3p mimic and pcDNA3.1-ITGB4 reversed the miR-302d-3p mimic-induced promoting effect or ITGB4 overexpression-stimulated suppressive role to the apoptotic ability of CHON-001 cells after LPS treatment (Figure 4C). Furthermore, apoptosis rate was attenuated by miR-302d-3p inhibitor but increased due to ITGB4 knockdown, which were all overturned by the cotransfection of miR-302d-3p inhibitor and si-ITGB4 (Figure 4D). Therefore, we concluded that the miR-302d-3p/ITGB4 axis may affect LPS-treated chondrocytes behaviors, including cell viability and apoptosis.

### Valproic Acid Treatment Decreases the Expression of miR-302d-3p but Elevates the Expression of ITGB4 in Osteoarthritis Cells

**FIGURE 5:**
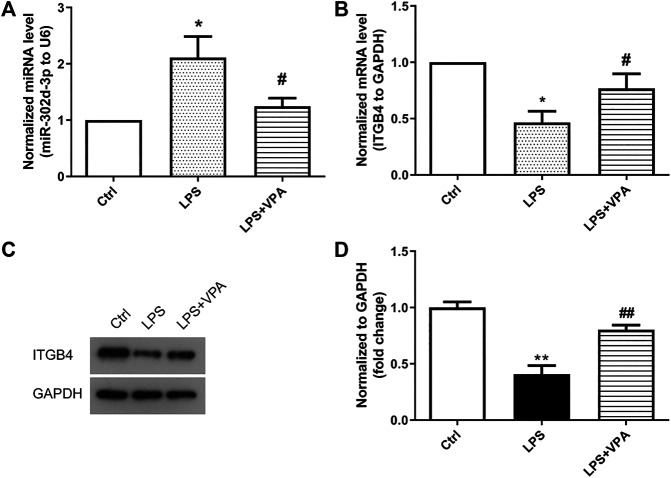
VPA treatment decreases the expression of miR-302d-3p but elevates the expression of ITGB4 in OA cells. **(A)** Effect of VPA treatment on the expression of miR-302d-3p in LPS-treated CHON-001 cells through qRT-PCR assay. *p < 0.05, ^#^ *p* < 0.05. **(B)** qRT-PCR analysis was used to determine the impact of VPA on the ITGB4 mRNA expression in OA cells. *p < 0.05, ^#^ *p* < 0.05. **(C,D)** The ITGB4 protein expression was examined using western blot and quantified via the normalization to GAPDH. **p < 0.01, ^##^ *p* < 0.01. Results are the average of three individual experiments in triplicate with error bars indicating mean ± SD (ANOVA followed by Tukey’s post hoc test).

In order to explore whether VPA is involved in the regulatory action of miR-302d-3p/ITGB4 axis in OA, we detected the expression of miR-302d-3p and ITGB4 in CHON-001 cells stimulated by LPS or/and VPA. The expression of miR-302d-3p was significantly increased by LPS. VPA treatment restored the expression of miR-302d-3p to the normal level (Figure 5A). Conversely, the mRNA and protein levels of ITGB4 were significantly decreased in LPS-treated CHON-001 cells, which was reversed by the stimulation of VPA (Figures 5B–D). Together, it can be assumed that VPA may exert a protective role via miR-302d-3p/ITGB4 in OA.

### Valproic Acid Prevents Chondrocytes from LPS-Induced Damage Through Regulating miR-302d-3p/ITGB4 Axis

**FIGURE 6:**
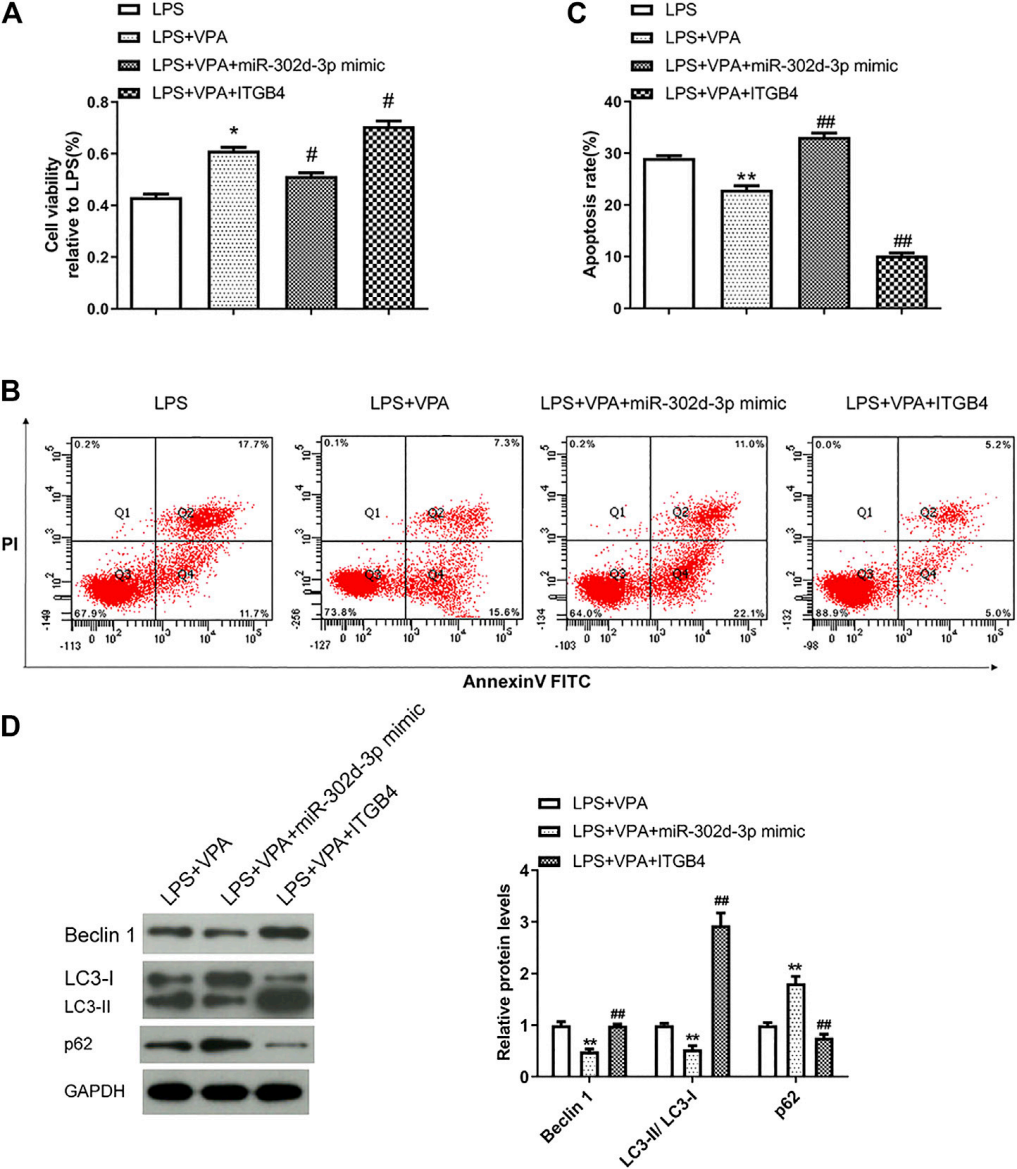
VPA prevents chondrocytes from LPS-induced damage through regulating miR-302d-3p/ITGB4. **(A)** Cell viability was measured by CCK-9 assay. *p < 0.05, ^#^ *p* < 0.05. **(B,C)** Flow cytometry assay showed the apoptosis rate of chondrocytes after various treatments and specific transfection. **p < 0.01, ^##^ *p* < 0.01. **(D)** Western blot analysis of autophagy-related proteins in CHON-001 cells transfected with miR-302d-3p mimic or pcDNA3.1-ITGB4 and then treated with LPS and VPA. **p < 0.01, ^##^ *p* < 0.01. Results are the average of three individual experiments in triplicate with error bars indicating mean ± SD (ANOVA followed by Tukey’s post hoc test).

The following experiments were designed and implemented to further clarify the interaction of VPA and miR-302d-3p/ITGB4 axis in OA cells. As shown in Figure 6A, the stimulatory effect of VPA was partially abolished by the miR-302d-3p overexpression but enforced by transfection of pcDNA3.1-ITGB4 in LPS-induced chondrocytes. Apoptosis assay manifested that overexpression of miR-302d-3p reversed the suppressive role of VPA on apoptotic capability, while overexpression of ITGB4 significantly strengthened the inhibitory impact induced by VPA treatment in an *in vitro* OA model (Figures 6B,C). The potential relevance of VPA and miR-302d-3p/ITGB4 in autophagy of OA cells was performed by western blotting. Compared with LPS + VPA group, upregulation of miR-302d-3p decreased the level of Beclin 1, retarded the conversion of LC3-I to LC3-II, and elevated p62 levels. However, in CHON-001 cells treated by LPS and VPA, ITGB4 overexpression promoted the level of Beclin 1 and accelerated the conversion from LC3-I to LC3-II but repressed p62 (Figure 6D). VPA could stimulate cell viability and autophagy but attenuate apoptosis rate in OA cells through regulating miR-302d-3p/ITGB4.

### Valproic Acid Treatment Inactivates the PI3K-AKT Signaling Pathway via Mediating miR-302d-3p/ITGB4 Axis in LPS-Treated Chondrocytes

**FIGURE 7:**
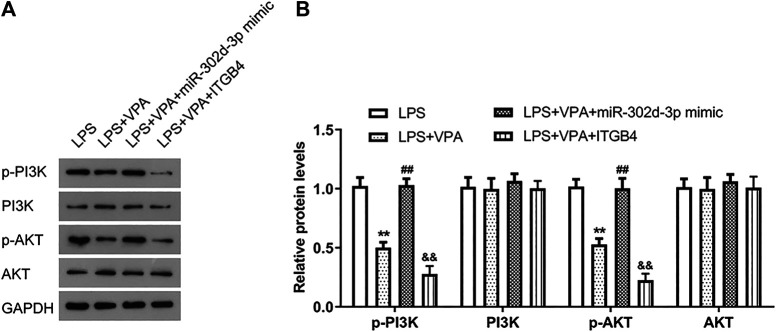
VPA treatment inactivates the PI3K-AKT signaling pathway via mediating miR-302d-3p/ITGB4 in LPS-treated chondrocytes. **(A)** Assessment of PI3K/p-PI3K and AKT/p-AKT levels in LPS-induced CHON-001 cells by western blotting. **(B)** Relative PI3K/p-PI3K and AKT/p-AKT levels were quantified and exhibited in column diagrams. **p < 0.01, ^##^ *p* < 0.01, ^&&^ *p* < 0.01. Results are the average of three individual experiments in triplicate with error bars indicating mean ± SD (ANOVA followed by Tukey’s post hoc test).

Considering that ITGB4 was selected as a gene enriched in the PI3K-AKT pathway, thus, western blot assays were conducted to decipher the effect of VPA/miR-302d-3p/ITGB4 on the activity of the PI3K-AKT pathway in OA cells. Figures 7A,B showed that compared with the LPS group, the levels of p-PI3K and p-AKT were markedly decreased in the LPS + VPA group. In OA cells induced by LPS, miR-302d-3p mimic rescued the inhibitory effect of VPA treatment on p-PI3K and p-AKT levels, whereas overexpression of ITGB4 intensified the suppressive role of VPA stimulation on the PI3K-AKT pathway. Thus, we speculated that VPA may inactivate the PI3K-AKT pathway possibly via regulating miR-302d-3p/ITGB4 in LPS-stimulated CHON-001 cells.

## Discussion

Osteoarthritis is a heterogeneous disease with complex etiologies, which mainly induces pain and disability in the elderly (Glyn-Jones et al., 2015; Wang et al., 2015). OA patients often reveal a broad range of clinical manifestations in response to treatment, posing a great challenge for the OA treatment (Zhu et al., 2018). Until now, there is no efficacious agent for the reversion of OA progression and modification of structure development.

In order to develop the new therapeutic strategies, we screened out DEGs in OA samples and found that ITGB4 expressed in OA with lower levels may be a potential therapeutic target. ITGB4 has been reported to be involved in multiple biological processes, such as cell proliferation, migration, and apoptosis (Li et al., 2019). Sopita et al. demonstrated that ITGB4 was expressed in chondrocyte sheets (Wongin et al., 2020). ITGB4 also regulates ECM that exerts a crucial role in the pathogenesis of OA (Zhong et al., 2020). In addition, we found that miR-302d-3p, directly targeting ITGB4, was expressed with higher levels in OA compared with normal controls. Downregulation of miR-302d-3p could promote proliferation and migration but suppress apoptosis in chondrocytes (Wang et al., 2019). Therefore, we speculated that miR-302d-3p/ITGB4 may play an important role in the progression of OA. Our analysis manifested that ITGB4 was significantly decreased in OA samples. Further *in vitro* experiments unearthed that ITGB4 promoted cell proliferation and inhibited apoptosis, while miR-302d-3p significantly inhibited cell viability and strengthened apoptosis of CHON-001 cells treated by LPS. These findings suggested that miR-302d-3p might aggravate the LPS-induced damage on CHON-001 cells via targeting ITGB4.

VPA is an HDAC inhibitor, which could be used to inhibit HDAC1 and induce proteasomal degradation (Zayed et al., 2011). Besides, the increased VEGF expression in chondrocytes might be associated with the destruction of articular cartilage. The induction of OA progression has also been reported to be related to high-dose VEGF (Zhu et al., 2018). A previous study has indicated that VPA functions as an inhibitor for the mRNA and protein expression of VEGF, VEGFR2, and bFGF (Phiel et al., 2001). Bevacizumab is an anti-VEGF antibody and widely utilized in inhibiting tumorigenesis, which could block the establishment of the OA *in vivo* model (Nagai et al., 2014). Another investigation has also confirmed that bevacizumab plays a repairing role in the articular cartilage of the rabbit knee OA model (Nagai et al., 2010). These research studies indicate that the VEGF inhibitor may shed new light on the development of effective drugs to treat OA. The main manifestations of OA include poor cell viability, increased apoptosis, and decreased autophagy (Hwang and Kim, 2015). Results revealed that VPA treatment could promote cell viability, attenuate apoptosis rate, and elevate autophagy of chondrocytes damaged by LPS. In addition, we also found that VPA induced the suppression of miR-302d-3p expression and elevation of ITGB4 expression in LPS-treated chondrocytes. However, whether VPA is implicated in the regulatory action of miR-302d-3p/ITGB4 in OA cells is unclear. Hence, we conducted an in-depth analysis and discovered that overexpression of miR-302d-3p alleviated the VPA-increasing cell viability, while ITGB4 enhancement intensified the stimulatory role of VPA to cell viability in LPS-treated chondrocytes. Apoptosis assay unveiled that VPA induced a decreased apoptosis rate, which was reversed by miR-302d-3p mimic and enhanced by ITGB4 overexpression. All findings suggested that VPA treatment remarkably decreased the level of miR-302d-3p and increased ITGB4 expression in the LPS-induced OA model, thereby relieving the injury of chondrocytes.

Additionally, our western blot results exhibited that VPA inhibited the activity of the PI3K-AKT pathway in the LPS-induced OA model by inhibiting the protein levels of p-PI3K and p-AKT, which was overturned by the transfection of miR-302d-3p mimic but intensified because of ITGB4 overexpression. This result indicated that VPA suppressed the development of OA via regulating the miR-302d-3p/ITGB4 pair and mediating the PI3K-AKT pathway.

Nevertheless, there are several limitations in the present study. On the one hand, it is difficult to represent physiological conditions in the current two-dimensional (2D) model. A three-dimensional (3D) tissue model of OA has been established, which retains the advantages of the 2D model and could mimic physiological conditions (Samvelyan et al., 2020). Moreover, *in vivo* experiments are required for more data validation. On the other hand, a previous report indicated that the use of VPA in clinics is related to the reduction of bone mineral density (BMD), which will increase the risk of OA (Arora et al., 2016). This is contrary to our current conclusion. Hence, further investigations should be performed to evaluate the clinical importance of VPA in OA. Finally, although VPA regulates the expression of miR-302d-3p/ITGB4, a direct or indirect effect needs to be carefully examined in the future.

## Conclusion

To summarize, our results revealed that VPA exerted an anti-OA role by regulating the miR-302d-3p/ITGB4 axis and stimulating the PI3K-AKT pathway. These findings might provide a new therapeutic avenue to treat OA.

## Data Availability

The original contributions presented in the study are included in the article/Supplementary Material; further inquiries can be directed to the corresponding author/s.

## Supplementary Material

Supplementary Figure 1Two replicates of western blot assay.
