# Supplementary figures and images for: Valproic Acid Protects Chondrocytes from LPS-Stimulated Damage via Regulating miR-302d-3p/ITGB4 Axis and Mediating the PI3K-AKT Signaling Pathway
Source: Front Mol Biosci. 2021 Apr 22;8:633315. doi: 10.3389/fmolb.2021.633315 (PMC8100442; doi:10.3389/fmolb.2021.633315)

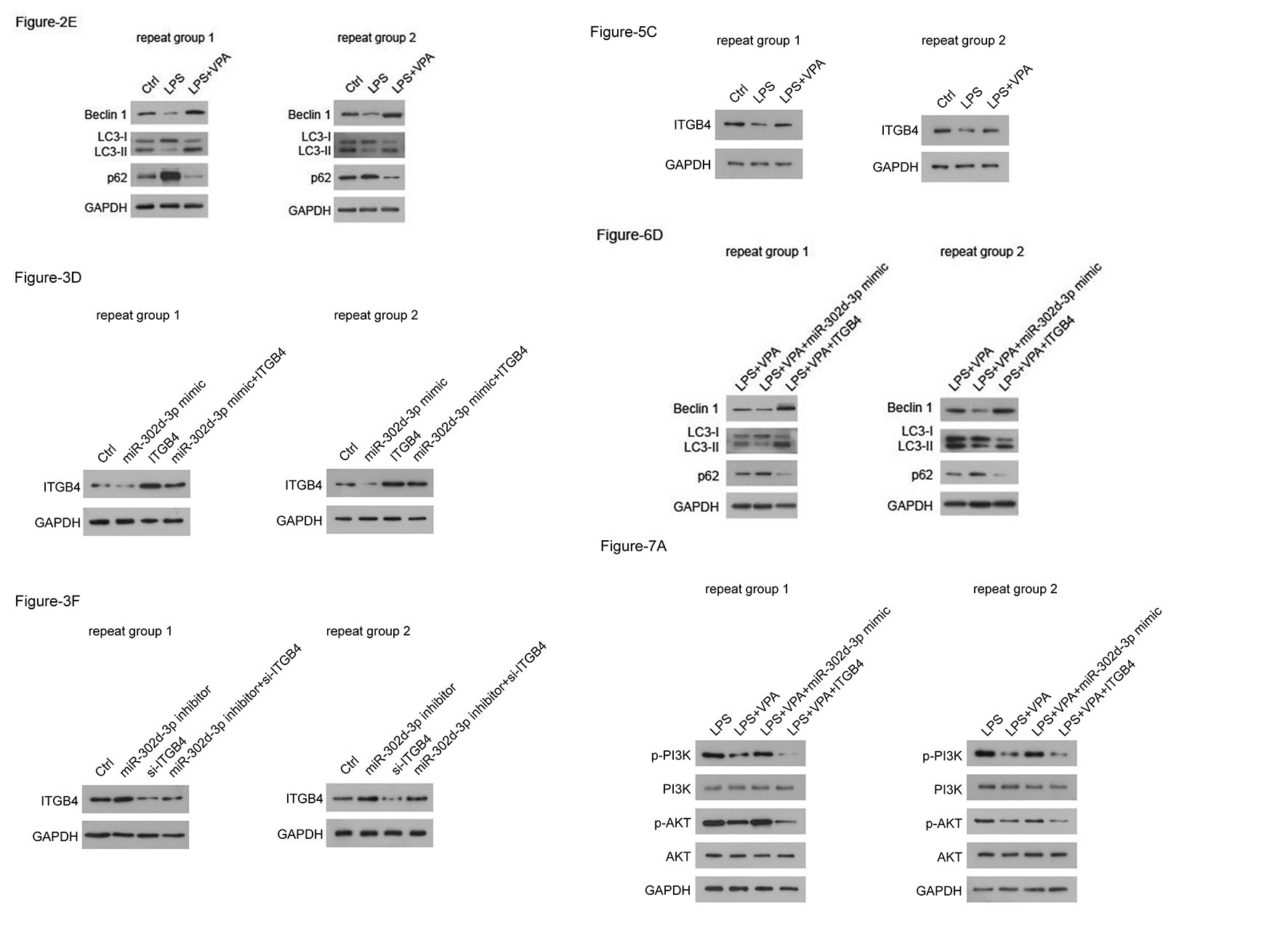

Supplement: Supplementary file 1 [file Image1.jpg]
